# The Medical Society of the State of New York

**Published:** 1894-03

**Authors:** 


BUFFALO MEDICAL AND SURGICAL JOURNAL
A MONTHLY REVIEW OF MEDICINE AND SURGERY.

*EDITORS:*
THOMAS LOTHROP, M. D. - - WM. WARREN POTTER, M. D.

All communications, whether of a literary or business character, should be addressed to the managing editor: 284 Franklin Street, Buffalo, N. Y.

Vol. XXXIII. MARCH, 1894. No. 8.

### THE MEDICAL SOCIETY OF THE STATE OF NEW YORK.

The eighty-eighth annual meeting was held in Albany, February 6, 7 and 8, 1894. Following our usual custom, we make such comment on the details of the meeting as the circumstances seem to warrant.

The society was fortunate in its presiding officer, Dr. Herman Bendell, of Albany, who conducted the duties of the chair with dignity and a clear understanding of the needs of the society. On Tuesday morning a less experienced officer might have become entangled in the meshes of parliamentary usage. When the question came up with reference to the nomination of State Medical Examiners, Dr. Bendell's position was so clearly right that the house would have sustained him overwhelmingly on the appeal of ex-President Pilcher, had the latter not exercised his great good sense in withdrawing it. The President's two addresses, inaugural and anniversary, were carefully-written and well-worded papers. The latter was especially rich in classic lore, and will take a high place in the annals of the society.

The papers announced on the program were of an unusually high scientific order, and showed the skilful joint work of the President and his able business committee. It required great tact and judgment to dispose of such a rich and extended program to the satisfaction of all concerned. The number of out-of-the-State guests who participated in the proceedings was unusually large and representative. Among the number were Dr. William H. Daly, of Pittsburg; Dr. Joseph Price and Dr. Charles P. Noble, of Philadelphia; Dr. E. W. Cushing, of Boston; Dr. George H. Rohé, of Baltimore; Dr. A. W. Johnstone, of Cincinnati; Dr. James F.

W. Ross, of Toronto; and Dr. Dudley P. Allen, of Cleveland. Dr. Ross has been a guest of the society heretofore, and his return gave occasion to his many friends to greet him with a cordiality that was as heartfelt as it was spirited. The same, too, may be said of Drs. Price and Cushing.

The dinner to the ex-presidents of the society, given by Dr. William C. Wey, of Elmira, at the Fort Orange Club, on Monday evening, February 5th, was a delightful occasion, and the guests were especially happy in their geniality and well-timed speeches. The host was at his best, which is all that need be said to those who are familiar with the ease and grace and genial *bon homme* of the veteran from Chemung.

This meeting, more than ever, demonstrated the propriety of separating the business and scientific proceedings, hence Dr. Albert Vander Veer, of Albany, wisely moved that the committee on by-laws be requested to consider this subject and report at the next meeting.

The interest in the several sessions was well maintained, even a large number remaining during the final sitting on Thursday morning.

The following officers and committees were chosen to serve for the ensuing year:

*President*, Dr. George Henry Fox, of New York; *Vice-President*, Dr. Frank S. Low, of Pulaski; *Secretary*, Dr. Frederic C. Curtis, of Albany; *Treasurer*, Dr. Charles H. Porter, of Albany.

*Committee of Arrangements*—Drs. William J. Nellis, Albany; Josiah Hasbrouck, Port Ewen; Reynold W. Wilcox, New York.

*Committee on By-Laws*—Drs. H. D. Wey, Elmira; A. R. Simmons, Utica; F. C. Curtis, Albany.

*Committee on Hygiene*—Drs. Charles E. Bruce, New York; H. R. Hopkins, Buffalo; A. N. Bell, Brooklyn; D. S. Burr, Binghamton; Lewis Balch, Albany; E. H. Loughran, Kingston; O. W. Peck, Oneonta.

*Committee on Legislation*—Drs. D. B. St. John Roosa, New York; Daniel Lewis, New York; D. V. O'Leary, Albany.

*Committee on Medical Ethics*—Drs. John S. Warren, New York; Charles Jewett, Brooklyn; Eugéne Beach, Gloversville.

*Committee on Prize Essays*—Drs. Franklin Townsend, Albany; A. Walter Suiter, Herkimer; Charles Stover, Amsterdam.

*Committee on Publication*—Drs. F. C. Curtis, Albany ; William Warren Potter, Buffalo ; F. D. Baily, Brooklyn ; C. H. Porter, Albany.

The next annual meeting of the society will be held in Albany, N. Y., February 5, 1895.

